# Corroborating molecular species discovery: Four new pine-feeding species of *Chionaspis* (Hemiptera, Diaspididae)

**DOI:** 10.3897/zookeys.270.2910

**Published:** 2013-02-18

**Authors:** Isabelle M. Vea, Rodger A. Gwiazdowski, Benjamin B. Normark

**Affiliations:** 1 Richard Gilder Graduate School, Division of Invertebrate Zoology, American Museum of Natural History, New York, NY, USA; 2 Graduate Program in Organismic and Evolutionary Biology, University of Massachusetts, Amherst, MA, USA; 3 Biodiversity Institute of Ontario, University of Guelph, Guelph, Ontario, Canada; 4 Department of Biology, University of Massachusetts, Amherst, MA, USA

**Keywords:** Armored scale insects, Diaspidini, Diaspidinae, cryptic species, endemic, North America, *Pinus*

## Abstract

The genus *Chionaspis* (Hemiptera, Diaspididae) includes two North American species of armored scale insects feeding on Pinaceae: *Chionaspis heterophyllae* Cooley, and *Chionaspis pinifoliae* (Fitch). Despite the economic impact of conifer-feeding *Chionaspis* on horticulture, the species diversity in this group has only recently been systematically investigated using samples from across the group’s geographic and host range. This paper provides morphological recognition characters for four new species that were recently hypothesized to exist on the basis of molecular evidence. The new species, here described, are *Chionaspis brachycephalon* Vea **sp. n.**, *Chionaspis caudata* Vea **sp. n.**, *Chionaspis sonorae* Vea **sp. n.** and *Chionaspis torreyanae* Vea **sp. n.**  One of the new species, *Chionaspis caudata* Vea, has a gland spine at the apex of the pygidium, between the median lobes, unlike any other species of *Chionaspis*. An identification key to the species of *Chionaspis* feeding on pine in North America is provided.

## Introduction

The armored scales insects (Hemiptera, Diaspididae) are a group of over 2500 described species of plant parasites (Ben-Dov et al. 2012). Adult females are characterized by a reduced morphology and a sessile habit on plant surfaces (Miller and Davidson 2005). Because armored scale insects are often cryptic in the field and are not susceptible to mass-collecting techniques, they are often overlooked in faunal surveys, and so new species tend to be discovered as agricultural pests (Evans et al. 2009, Wolff and Claps 2010, Dones and Evans 2011). Conventionally, new species of armored scales are discovered based on unique combinations of morphological characters.

However, some shortcomings of conventional morphological species descriptions are their reliance on specimens sampled from a limited number of locations, or hosts, as is often the case when identifying agricultural pests. Such limited sampling may fail to observe a range of intraspecific morphological variation across hosts and geography. More importantly, conventional species descriptions of armored scales are not often corroborated with genetic measures of species boundaries (but see Evans et al. (2009) and Rugman-Jones et al. (2009)). This reliance on morphology alone may obscure the nature of species diversity in armored scales. Some putative species, identified only by molecular markers, may be cryptic species (Bickford et al. 2007) - which show no interspecific morphological variation (Andersen et al. 2010, Gwiazdowski et al. 2011). Because conventional, morphological criteria for species differences may fail to distinguish new species, Gwiazdowski et al. (2011) undertook a molecular study of species diversity of *Chionaspis* collected across North America from 54 species of *Pinus*. Here we report a parallel morphological study of species diversity in this same sample.

Current taxonomy of the genus *Chionaspis* Signoret recognizes two pine-feeding species, *Chionaspis heterophyllae* Cooley and *Chionaspis pinifoliae* (Fitch). These species are native to North America (Watson 2005), and are considered pests on *Pinus* in forests and ornamental settings (Miller 1996, Miller and Davidson 2005). *Chionaspis pinifoliae* has been recognized as a pest for over 150 years and has been a subject of at least 189 publications. *Chionaspis heterophyllae* has been a subject of at least 55 publications (Veilleux et al. 2011), and together, the two species have been the focus of three PhD dissertations (Nielsen 1970, Shour 1986, Gwiazdowski 2011).

*Aspidiotus pinifoliae* was first described by Fitch as a pest of pines, “which fixes itself upon the leaves, exhausting them of their juices and then causing them to perish and fall, and the end of the limbs to die when thus defoliated” (Fitch 1956: 488). Fitch described the species based on specimens on pine needles that were sent to him from Robert W. Kennicott who collected them in the “yard of S. Francis, Esq. in the city of Springfield” in Illinois. Fitch describes the arrangement of the scale insects on the pine needle as well as their general shape and color, but the pine species was not identified and the scale insects were never prepared and mounted on a slide. Nevertheless, the series of specimens on needles used for the description were found at the New York State Agricultural Society (New York State Museum). This should allow the designation of a lectotype for *Chionaspis pinifoliae* (not treated here). The species was subsequently placed in five different genera (*Mytilaspis pinifoliae*, LeBaron 1872: 83; *Chionaspis pinifoliae*, Comstock 1881: 318; *Leucaspis pinifoliae*, García Mercet 1912: 215; *Chionaspis (Phenacaspis) pinifoliae*, Balachowsky 1930d: 266; *Polyaspis pinifolii*, Lindinger 1935: 140; *Phenacaspis pinifoliae*, Ferris 1937: SI-93.)

*Chionaspis pinifoliae heterophyllae* was first described by Cooley in 1897 (1897: 281-282) from specimens collected in Florida (Cooley 1899). Cooley’s original description distinguished this subspecies (which he called a variety) from *Chionaspis pinifoliae* by its smaller body size and more rounded and less conspicuous median lobes (Cooley 1897, Andresen 1957). The differences in these characters were later illustrated, on plate 7, of Cooley’s monograph of *Chionaspis* (Cooley 1899). Subsequently, *Chionaspis pinifoliae heterophyllae* has undergone two taxonomic changes; the first in rank to full species by the name *Phenacaspis heterophyllae*, (MacGillivray 1921:347), and the second by reassignment to *Chionaspis* as *Chionaspis heterophyllae*, (Borchsenius 1966: 122).

Since 1921 (MacGillivray 1921) taxonomists have only recognized two pine-feeding *Chionaspis* (previously *Phenacaspis*) in North America, but the recent reanalysis of species diversity within this group by Gwiazdowski et al. (2011) indicated the presence of at least 10 closely related species feeding on pine. The methods of Gwiazdowski et al. (2011) used multi-locus genealogical concordance to delimit species. This method is expected to be conservative because it should only detect species boundaries that are old and impermeable enough for monophyly to have evolved at a majority of loci (Neigel and Avise 1986, Hudson and Coyne 2002).

Most of the specimens collected by Gwiazdowski et al. (2011) are morphologically indistinguishable from *Chionaspis pinifoliae* or *Chionaspis heterophyllae*. The few specimens that could be distinguished from both *Chionaspis pinifoliae* and *Chionaspis heterophyllae* could be placed in five morphological groups, and these groups would be recognized as species by conventional morphological criteria (Miller and Davidson 2005, Watson 2005). Only one of these four morphological groups was recognized as a species by Gwiazdowski et al. (2011), whereas the other four morphological groups were subsumed within more inclusive undescribed species. All morphological groups are tightly correlated with geography, host affiliation or both (see Gwiazdowski et al. (2011) and species descriptions below).

These results highlight the possibility that even species that are well known, as in the case of pest species, may be more diverse than previously thought (i.e. contain cryptic species). It is not immediately clear how best to assign taxonomic status to cryptic species (but see Cook et al. (2010)), and so here we provide morphological descriptions for four new species of *Chionaspis* which are distinguished in the analyses of Gwiazdowski et al. (2011) as belonging to clearly diverged lineages, and possessing a unique combination of morphological characters distinct from both *Chionaspis pinifoliae* and *Chionaspis heterophyllae*.

## Materials and methods

Field collection and slide mounting of all specimens were accomplished using the protocols described by Gwiazdowski et al. (2011). While some species of *Chionaspis* can show a tissue-specific morphology -- where broodmates developing on different plant tissues (e.g. leaves or bark) have very different morphology (Takagi 1985, Liu et al. 1989) -- specimens from Gwiazdowski et al. (2011) were all collected from the same host tissue: pine needles. Illustrations were made by hand using a camera lucida on a Zeiss 47 46 20-9900 microscope and an Olympus CHBS, and digitally edited with Photoshop CS4 14.0.0. They follow the convention used in scale insect illustration, with each figure displaying the dorsal body surface on the left side and the ventral body surface on the right side. Enlargements of significant features are located around the body. The morphological terminology and measurements in the descriptions below follows the conventions of Miller and Davidson (2005). In brief, abbreviations in the text refer to different pygidial lobes (trullae of Takagi): L1 for the median lobes, L2 for the second pair of lobes, L3 for the third pair of lobes and L4 for the fourth pair of lobes. Formulas are provided for the number of gland spines and microducts present between the pygidial lobes. For example, 1-1-1 indicates 1 gland spine in the first space (between L1 and L2); one in the 2nd space (between L2 and L3); and one in the 3rd space (between L3 and the position where L4 would be). Occasionally, the number of microducts subtending gland spines differs from the number of gland spines, and the microduct formula (microducts subtending gland spines in the 1st-2nd-3rd spaces) is indicated in parentheses. Length measurements are given as ranges with the median value in parentheses. The distance between the median lobes is measured from the medial margins, at the midpoint between base and apex. The species described here correspond to the morphological groups indicated by the letters B, C, D, and E in Figures 2 through 4 from Gwiazdowski et al. (2011). Here, each species name is followed by the corresponding morphogroup letter from Gwiazdowski et al. (2011).

Slide mounted type specimens have been deposited at the National Insect Collection, Instituto de Biología, Universidad Nacional Autónoma de México, Mexico City (CNIN), the United States National Entomological Collection (Coccoidea collection) at the U.S. National Museum of Natural History (USNM), USA and the University of Massachusetts Insect Collection, Amherst, MA, USA (UMAM). Genomic DNA from all types, as well as lots supplying the type material (additional specimens in-situ on host tissue) from the study of Gwiazdowski et al. (2011), has been deposited at the American Museum of Natural History’s Ambrose Monell Cryo Collection (AMCC). The DNA sequences used by Gwiazdowski et al. (2011) comprise three loci: the D2-D3 portion of the large ribosomal subunit rDNA (28S), elongation factor 1-alpha (EF-1a), and a mitochondrial fragment spanning parts of cytochrome C oxidase subunits I and II (COI-COII). Sequences for these three loci, for all type specimens, have been deposited in GenBank (Benson et al. 2012). The repository, associated accession numbers, and locality information for all type material and material with published DNA sequences is provided in the species description and Appendix 1and 2. The USNM and CNIN do not assign accession numbers to type specimens; types are incorporated within the general Coccoidea collections, and these are arranged alphabetically by family, genus, species. Holotype specimens are prominently marked in red, additionally CNIN paratypes are marked in green.

## Taxonomy

### Chionaspis brachycephalon

Vea sp. n.

urn:lsid:zoobank.org:act:45770622-189F-47C8-A6B3-369B5BDDF3EB

http://species-id.net/wiki/Chionaspis_brachycephalon

Figure 1

#### Morphogroup D in Gwiazdowski et al. (2011).

#### Type material.

**Type locality:** Mexico, Durango state, Navios, 23°53.95'N, 105°2.83'W, on needle of *Pinus cooperi* Blanco, 24 September 2007, R. Gwiazdowski and A. Garcia Arévalo coll.

#### Type specimen:

Holotype adult female, slide-mount in balsam. Original label: “D1765A, Mexico, Durango, Resturante “Los Pinos”, Navios, 1.ix.2007, 23°53' 56.9"N, 105°02'49.6"W, R. Gwiazdowski, A. Garcia Arévalo, *Pinus cooperi* ”, deposited at CNIN.

Paratype: Adult female, slide-mount in balsam. D1765B, same collection data as holotype, deposited at USNM.

Other material examined: Adult female, slide-mount in balsam. Original label “D1718A, Mexico: Mexico, Hwy 95 South of Tres Marias, 1.ix.2007, 19°01'37.5"N, 99°12'35.2"W, R. Gwiazdowski, D. Gernandt, *Pinus pseudostrobus* Lindl.”, deposited at UMAM. Adult females on separate slides, D1718C, D and F, same collection data as D1718A, deposited at CNIN.

#### Diagnosis.

*Chionaspis brachycephalon* Vea differs from other *Chionaspis* by the following combination of characters (Table 1): small head, gland spine formula variable from 1-1-1 to 2-2-2 (median: 2-2-2), microduct formula also variable from 2-2-2 to 3-3-4 (median: 3-2-2); numerous marginal gland spines on abdominal segments 3 to 5, absent from abdominal segment 1 and 2; variable number of notches present on all pygidial lobes.

#### Description.

**Field characters:** All pine-feeding *Chionaspis* discussed here, including *Chionaspis heterophyllae* and *Chionaspis pinifoliae*, are indistinguishable by eye in the field. The adult female for all species possesses a white oystershell-shaped and slightly convex cover, with the amount of posterior expansion varying according to the diameter of host needles. Body elongate, color varying from yellow when immature to reddish brownish in specimens containing eggs, with lateral protrusion on the anterior abdominal segments. Found on needles.

**Figure 1.**
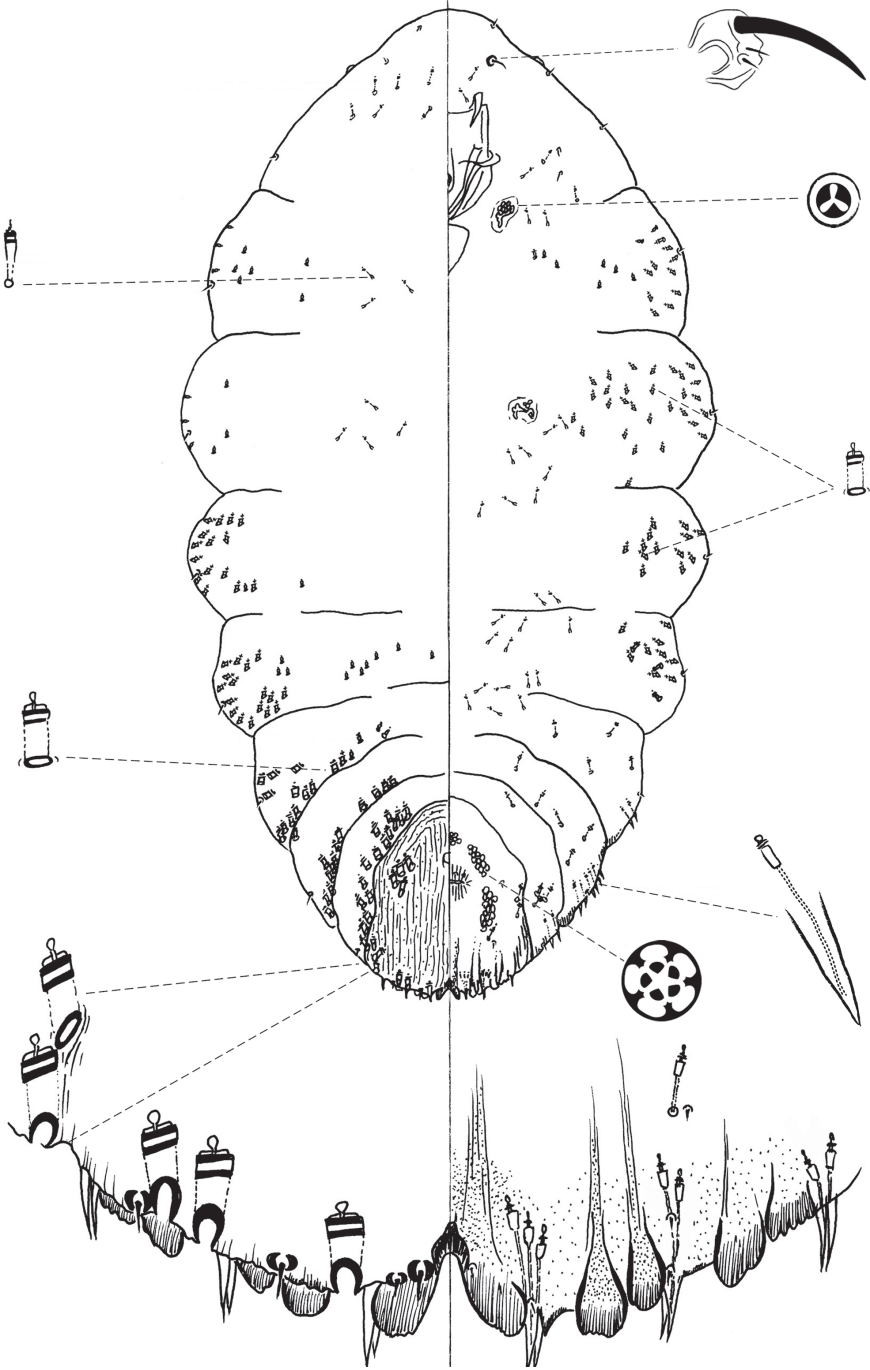
*Chionaspis brachycephalon* Vea sp. n., adult female.

**Slide-mounted adult female** (Figure 1), broadest at metathorax, with thoracic segments lobed laterally, prothorax becoming narrower towards the anterior, ending with a pointed head, giving the appearance of a reduced, shrunken head; length of holotype 1.33 mm, range (n= 6) 0.85 – 1.33 mm; maximum width of holotype: 0.63 mm; range (n=6) 0.45 – 0.66 mm.

**Pygidium: Lobes**. Posterior margin with 3 pairs of definite lobes (L1, L2 and L3), fourth pair (L4) appearing as series of low, sclerotized points; paraphyses absent. L1 separated by space 0.31 – 0.6 (0.4) times width of lobes, with a heavily sclerotized yoke, lateral and medial margins of L1 diverging from base to apex, medial margin convex with notches towards apex, lateral margin entire; L2 bilobed, smaller than L1, medial lobule larger than lateral, sometimes with notches, lateral lobule minutely notched; L3 bilobed, lateral lobule shorter than medial lobule, with minute notches, in some specimens appearing membranous or obsolete. **Gland spines.** Gland spine formula varying from 1-1-1 to 2-2-2 (2-2-2) (microduct formula varying from 2-2-2 to 3-3-4 (3-2-2)), gland spines projecting beyond L1; with 1 – 2 (2) gland spines on abdominal segment 5; without gland spines between L1. **Ducts.** Large macroducts in submedian area of segments 5 and 6 (with 5 – 10 (8) on segment 5 and 4 – 7 (5) on segment 6); in submarginal areas of segment 5 (with 8 – 10 (9)); marginal area of segments 5 to 7 (with 1 on segment 7, 2 on segment 6, 2 – 3 (2) on segment 5); absent on segment 8. Largest macroduct on segment 7 (between L1 and L2) 15 – 17.5 (17.5) μm long. Pygidial microducts always on venter in submarginal areas of segment 5 to 7, with 2 – 4 (2) ducts on segment 5, 1 – 3 (2) ducts on segment 6 and 1 ducts on segment 7 ; pygidial microduct absent from dorsum. **Pores.** Perivulvar pores with 5 loculi, in 5 groups, 1 median with 9-15 (13) pores, 2 anterolateral with 19 – 33 (23) pores, 2 posterolateral with 19 – 33 (25) pores. **Anal opening.** Located 6.8 – 10.2 (8.4) times length of anal opening from base of median lobes, diameter 15 – 20 (17.5) μm.

#### Setae.

Dorsal setae: 2 setose on L1, 1 setose (15 μm) between lobules of L2 and L3 lobes. Ventral setae: 1 small on median lobe, 1 marginal at base of each gland spine cluster and 1 in submarginal area of each segment, 2 in submedian area of segment 6, half as long as dorsal setae; 2 pairs of setae in a row anterior to the vulva**.**

**Prepygidium: Gland spines.** Near each body margin on segments 3 and 4, absent from segment 1 and 2; with 2 – 6 (4) on segment 3 and 2–5 (4) gland spines on segment 4, all protruding from margin..**Ducts**. Macroducts of 2 sizes; large macroducts in submedian and submarginal areas of abdominal segments 3 and 4. Small macroducts in submedian area of any or all of segments 2 to 4, and in marginal areas from meso- or metathorax to segment 3. Prepygidial microducts present on venter and dorsum from segment 1 to 4, sparsely distributed.

**Cephalothorax:** Microducts present on venter and dorsum with a slight concentration around thoracic spiracles.Perispiracular pores with 3 loculi, anterior spiracles with 3 – 5 (5) pores, posterior spiracles with 1 – 5 (2) pores. Eyes represented by small sclerotized area, located on body margin at level near anterior clypeolabral shield. Antennae each with 1 long seta and 2 minute setae, distance between antennae 42.5 – 90 (57.5) μm.

**Etymology**. The epithet *brachycephalon* is a noun, derived from Greek, meaning “short head”, from *brachy*- short + *cephalon* head. The epithet refers to the head shape of this species, which appears smaller than that of other pine-feeding *Chionaspis*.

**Table 1.** Diagnostic morphological characters for six species of pine-feeding *Chionaspis*.

**Features**	***Chionaspis pinifoliae (Fitch)***	***Chionaspis heterophyllae* Cooley**	***Chionaspis brachycephalon* Vea, sp. n.**	***Chionaspis caudata* Vea, sp. n.**	***Chionaspis sonorae* Vea, sp. n.**	***Chionaspis torreyanae* Vea, sp. n.**
Margins of prothorax	slightly convergent towards anterior	slightly convergent towards anterior	sharply convergent towards anterior	slightly convergent towards anterior	slightly convergent towards anterior	slightly convergent towards anterior
Gland spine formula	1-1-1	1-1-1	2-2-2	2-2-1	1-1-1	2-2-2
Microduct formula	1-1-1	1-1-1	3-2-2	2-2-1	1-1-1	2-2-2
Shape of the median lobes (L1)	basally slightly diverging, then parallel sided	diverging throughout	diverging throughout	parallel sided	medial margin parallel sided to mid margin then diverging	basally slightly diverging, then parallel sided
Gland spine between L1	0	0	0	1	0	0
Gland spines on segment 5	1	1	1 – 2 (2)	0	1	1
Gland spines on segment 4	1 – 3	1 – 3	2–5 (4)	1 – 2 (1)	1 – 4 (2)	1 – 2 (2)
Gland spines on segment 3	2-7	2-5	2 – 6 (4)	1 – 7 (4)	4 – 7 (5)	2 – 4 (3)
Submedial macroducts on segment 6	2 – 6 (3)	2 – 4 (3)	4 – 7 (5)	3 – 4 (3)	4 – 5 (5)	3 – 6 (4)
L1 margin	Entire or medial notches	Lateral and medial notches	Medial notches	Entire (rarely notched)	Notches on diverging part of medial margin	Entire (rarely one notch)
L2 margin	Entire	Entire or with a few small notches	Sometimes notched	Medial lobule slightly notched, lateral lobule entire	Entire	Entire
L3	Entire	Inner lobule entire or with a few small notches, outer lobule strongly notched	Inner lobule with minute notches, outer lobule notched and obsolete	Medial lobule entire, lateral lobule recessed, and notched	Entire or with slight notches	Entire

### Chionaspis caudata

Vea sp. n.

urn:lsid:zoobank.org:act:CEE3C93A-372F-40B2-A1E1-B778BC1500F4

http://species-id.net/wiki/Chionaspis_caudata

Figure 2

#### Morphogroup C in Gwiazdowski et al. (2011).

#### Type material.

**Type locality:** Mexico, Oaxaca state, Oaxaca, 16°11.99'N, 96°31.52'W, on needle of *Pinus patulata longipedunculata* (Loock ex Martínez), 28 August 2007, R. Gwiazdowski and M. Dahlberg coll.

#### Type specimens:

Holotypeadult female, slide-mount in balsam. Original label “D1703D Mexico: Oaxaca, Oaxaca, Hwy 175, 28.viii. 2007, 16°11'59"N, 96°31'30.9"W, R. Gwiazdowski, M. Dahlberg, *Pinus patulata longipedunculata*”, deposited at CNIN.

Paratypes: adult female, slide-mount in balsam, D1703A, same information as the holotype, deposited at USNM. Adult female, slide mount in balsam, D1703G, same collection data as the holotype, deposited at USNM. Adult females on separate slides, D1703F, H, I, J, K and L, same information as D1703D, deposited UNAM for all but D1703F, deposited at CNIN.

Other material examined: Adult female, slide mount in balsam. Original label “D1702A, Mexico: Oaxaca, Oaxaca, Hwy 175, 28.iii.2007, 16°10'31.3"N, 96°30'24.2"W, R. Gwiazdowski and M. Dahlberg, *Pinus pseudostrobus oaxacana*”, deposited at UMAM. Adult female, slide mount in balsam. Original label “D2275C, Mexico, Xalapa, HWY 131, ~2.6 km N of Atzalan, 15.i.2009, *Pinus chiapensis*”; adult female, slide mount in balsam. Orginal label “D2292C, Mexico, Guerrero, ~30 km, North E of Atoyac de Alvarez along road perpendicular to HWY 200, 20.i.2009, *Pinus chiapensis*”; adult female, slide mount in balsam, D2292D, same collection data as D2292C; adult female, slide mount in balsam. Original label “D2296B. Mexico, Chiapas, ~13.5 km North of Chamula, 24.i.2009, *Pinus chiapensis*”; adult female, slide mount in balsam, D2296C, same collection data as D2296B; all additional material deposited at UMAM.

#### Diagnosis.

*Chionaspis caudata* Vea differs from other *Chionaspis* with the following combination of characters (Table 1): median lobes (L1) unyoked, parallel-sided, with a single gland spine between them, subtended by a microduct; submedian microducts absent on abdominal segment 7; gland spine absent on abdominal segment 5; head square-shaped, body with an extended thorax relative to other pine-feeding *Chionaspis*.

#### Description.

**Field characters:** All pine-feeding *Chionaspis* reported here, including *Chionaspis heterophyllae* and *Chionaspis pinifoliae* are indistinguishable by eye in the field. See the description above for *Chionaspis brachycephalon* Vea.

**Figure 2.**
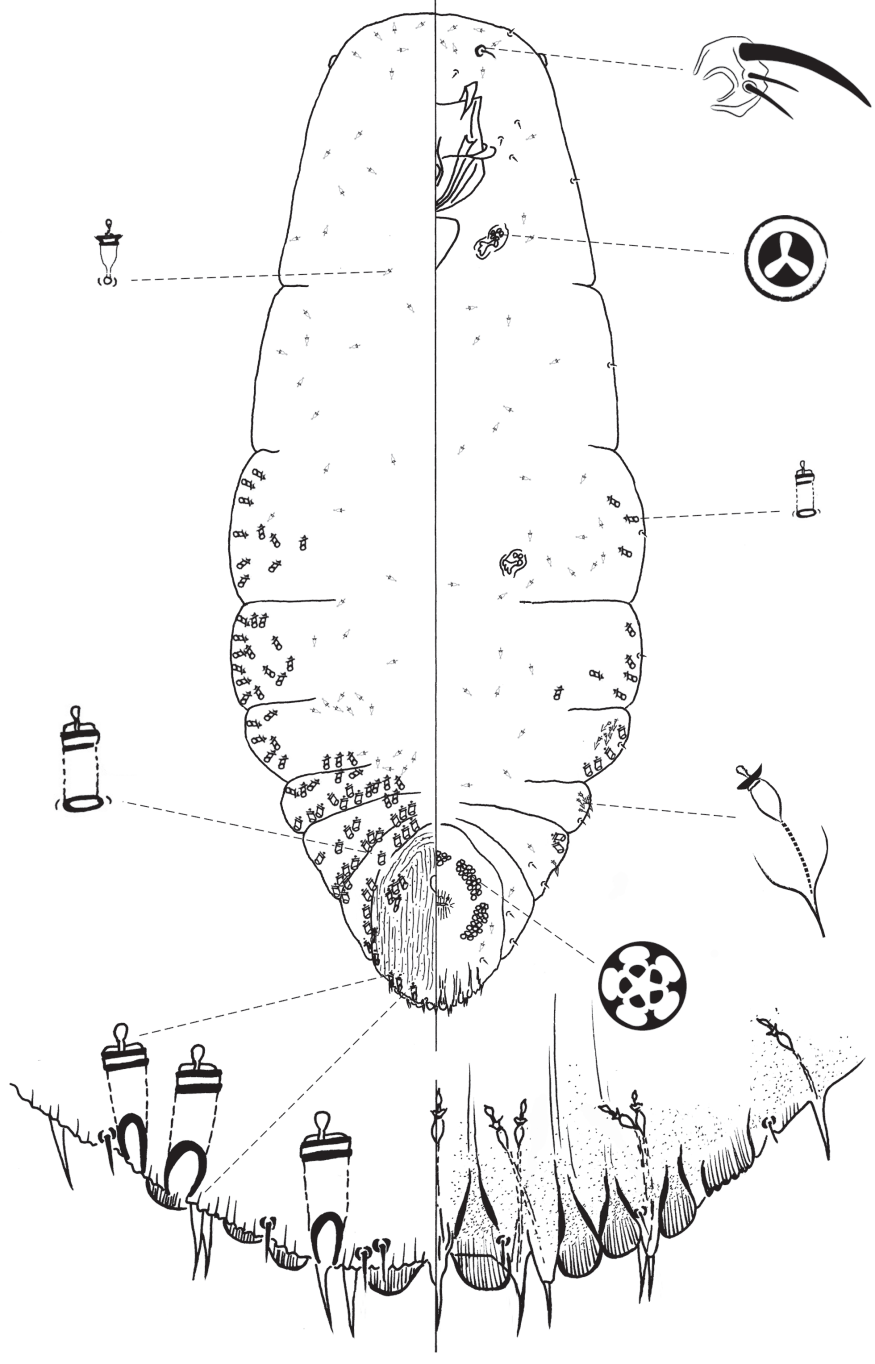
*Chionaspis caudata* Vea sp. n., adult female.

**Slide-mounted adult female** (Figure 2), spindle-shaped and elongate, slightly lobed to parallel-sided laterally; length of holotype 1.75 mm, range (n= 11) 1.38 – 2.03mm; maximum width of holotype: 0.61 mm; range (n=11) 0.48 – 0.7mm.

**Pygidium:** **Lobes**. Posterior margin with 3 pairs of lobes (L1, L2 and L3), fourth pair (L4) appears as series of low, sclerotized points; paraphyses absent. L1 separated by a space 0.6 – 1 (0.73) times width of lobes, without a yoke, lobes completely separated, lateral margins parallel-sided, entire, rarely notched; L2 bilobed, smaller than L1, lobules subequal, inner lobule slightly notched, outer lobule entire; L3 bilobed, medial lobule similar to L2, lateral lobule recessed and serrated. **Gland spines**. Gland spine formula varying from 1-1-1 to 3-3-2 (2-2-1) (microduct formula varying from 1-1-1 to 3-3-1 (2-2-1)), with always 1 gland spine between L1, subtended by 1 microduct; gland spine on segment 5 always absent. Gland spine microduct slender with a relatively developed collar at apex. **Ducts**. Large macroducts in submedian area of segments 5 and 6 (with 4 – 6 (6) on segment 5 and 3 – 4 (3) on segment 6); in submarginal areas of segment 5 (with 4 – 8 (7) macroducts); marginal area of segments 5 to 7 (with 1 on segment 7, 2 – 3 (2) on segment 6, 2 – 3 (2) on segment 5); absent on segment 8. Largest macroduct on segment 7 (between L1 and L2) 15 – 22.5 (20) μm long. Pygidial microducts always on venter in submarginal areas of segment 5 and 6, with 1 – 2 (2) duct on segment 5 and 2-3 (2) ducts on segment 6, always absent from segment 7; pygidial microducts absent from dorsum. **Pores**. Perivulvar pores with 5 loculi, in 5 groups, 1 median group with 10 – 15 (13) pores, 2 anterolateral groups with 23 – 27 (25) pores, 2 posterolateral groups with 18 – 27 (24) pores. **Anal opening**. Diameter 15 – 22.5 (17.5) μm, located 6.7 – 11.7 (9.9) times length of anal opening from base of median lobes. **Setae.** Dorsal setae: 2 setose on L1, 1 spinose between lobules of L2 and L3. Ventral setae: 1 small on L1, 1 marginal at base of each gland spine cluster and 1 submarginal area of each segment, 2 on submedian aerea of segment 6, half as long as dorsal setae; 2 pairs of setae in a row anterior to the vulva.

**Prepygidium:** **Gland spines**. Near each body margin from segment 1 or 2 to 4, with 0 – 4 on segment 1, 0 – 5 (4) on segment 2, 1 – 7 (4) on segment 3 and 1 – 2 (1) gland spines on segment 4, which are short and protrude from the margin. Gland spines from segment 1 to 3 are the smallest, and never protrude from the margin. **Ducts.** Macroducts of 2 sizes; largest macroducts in submedian areas of abdominal segments 4 and 3. Small macroducts in submedian area of segments 3 and 4, and in submarginal areas of segments 1 to 4. Prepygidial microducts present on venter from segment 1 to segment 3, in marginal or submarginal areas from head to segments 2 to 3. Prepygidial microducts on dorsum on segments 1 to 4, often in conspicuous clusters submedially.

**Cephalothorax:** Small macroducts present on last thoracic segment, marginally and submarginally. Microducts present on both surfaces, evenly distributed. Perispiracular pores primarily with 3 loculi, anterior spiracles with 6 – 8 (7) pores, posterior spiracles with 2 – 3 (2) pores. Eyes represented by small sclerotized area, located on body margin at level near anterior clypeolabral shield. Antennae each with 1 long seta. Distance between antennae 122.5 – 375 (135) μm.

#### Etymology.

*Chionaspis caudata* Vea possesses an unusual median gland spine between the median lobes. The epithet *caudata* is a Latin adjective meaning tailed (caudate), derived from *cauda*, tail, and referring to this peculiar feature.

#### Notes.

*Chionaspis caudata* Vea differs from the other species by the rather square-shaped head and noticeably longer body, the presence of a single gland spine subtended by one microduct between the median lobes, and the gland spine formula. The presence of the median gland spine is striking as this feature prevents this species from keying to the genus *Chionaspis* (or indeed any related genus) in available keys to genera; however, the phylogenetic analyses of Gwiazdowski et al. (2011) unambiguously place *Chionaspis caudata* Vea within *Chionaspis*.

### Chionaspis sonorae

Vea sp. n.

urn:lsid:zoobank.org:act:B04204CC-3A4F-4BC2-9BB3-25A3CAC95C44

http://species-id.net/wiki/Chionaspis_sonorae

Figure 3

#### Morphogroup B in Gwiazdowski et al. (2011).

#### Type material.

**Type locality.** Mexico, Sonora state, Tecora, 28°22.45'N, 108°56.11'W, on needle of *Pinus engelmannii* Carr, 8 October 2007, R. Gwiazdowski, T.R. Van Devender and A. Lilia Reina coll.

**Type specimens:** Holotype adult female, slide-mount in balsam. Original label “D1781A, Mexico: Sonora, Yecora, 8.x.2007, 28°22'26.8"N, 108°56'06.3"W, R. Gwiazdowski, T. R. Van Devender, L.Van Devender, *Pinus engelmannii* (Carr.)”, deposited at CNIN.

Paratypes: Adult females on separate slides, D1781C and F same collection data as D1781A; D1781C deposited at CNIN and D1781 F deposited at UMAM.

Other material examined: Adult female, original label “D1780A, Mexico, Sonora, West of Yecora, 7.x.2007, 28°21'33.5"N, 109°01'48.3"W, R. Gwiazdowski, T. R. Van Devender, L. Van Devender, Pinus engelmannii (Carr.)”, deposited at USNM. Adult females on separate slides, D1780B, C, D, E, F and G, same collection data as D1780A, deposited at UMAM.

#### Diagnosis.

*Chionaspis sonorae* Vea is distinguishable from other *Chionaspis* by the combination of the following characters (Table 1): median lobe shape unusual, broad, medial margins parallel or slightly convergent in basal half, abruptly angled near midpoint, with distal half divergent, serrated; yoke horseshoe-shaped; microducts sparse.

#### Description.

**Field characters:** All pine-feeding *Chionaspis* reported here, including *Chionaspis heterophyllae* and *Chionaspis pinifoliae* are indistinguishable by eye in the field. See the description above for *Chionaspis brachycephalon* Vea.

**Figure 3.**
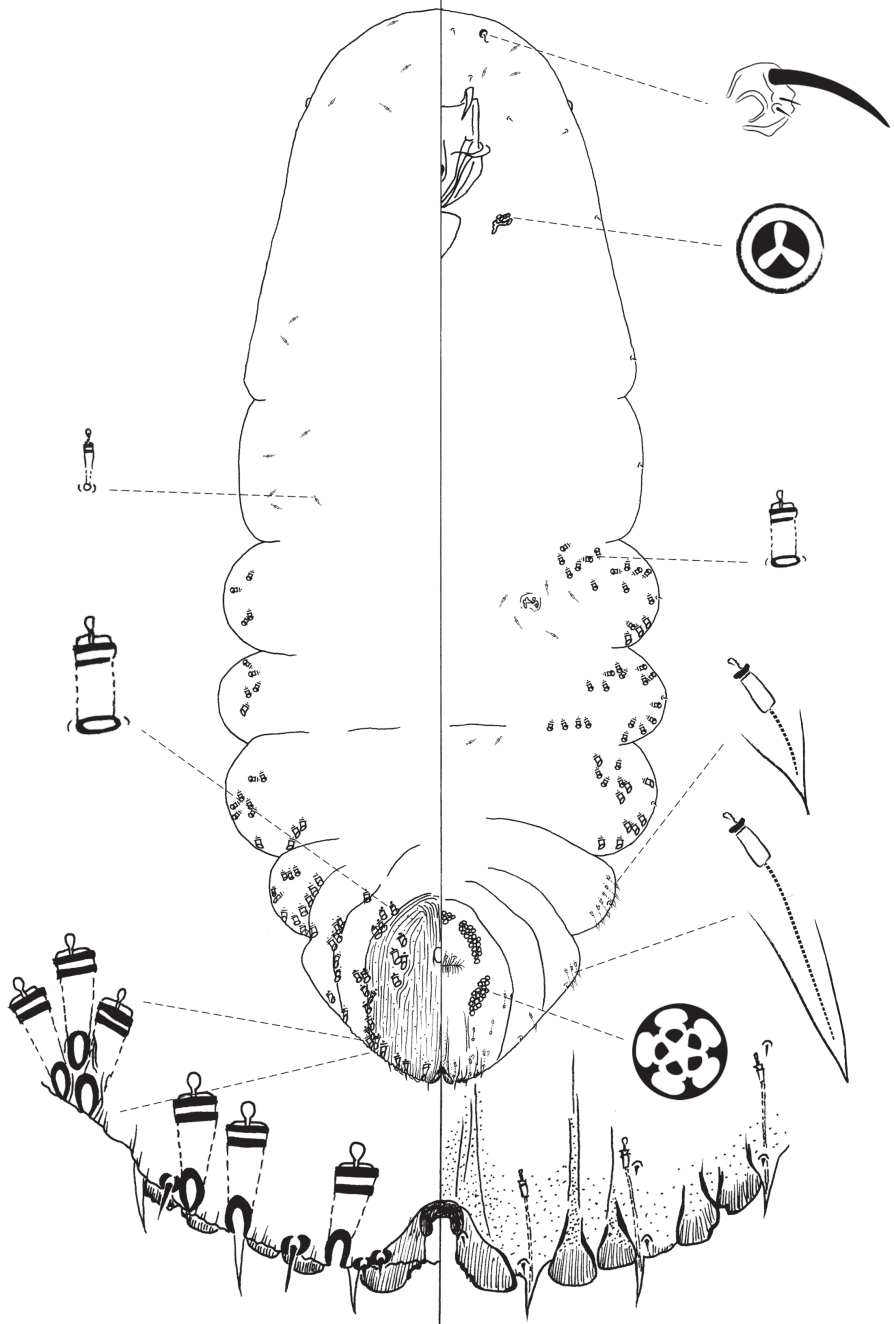
*Chionaspis sonorae* Vea sp. n., adult female.

**Slide-mounted adult female** (Figure 3) spindle-shaped and elongate, lobed laterally and broader posteriorly (broadest at metathorax or abdominal segment 1), length of holotype 1.29 mm, range (n=10) 1.29 – 1.83 mm; maximum width of holotype: 0.59 mm; range (n=10) 0.59 – 0.7 mm.

**Pygidium:** **Lobes**. Posterior margin with 3 pairs of definite lobes, fourth pair of lobes appearing as series of low, sclerotized points; paraphyses absent. L1 separated by space 0.3 times width of lobes, with a horseshoe-shaped yoke, lateral margins of lobes divergent, medial margin parallel from the base to midpoint, then diverging in apical half (with notches on diverging part); L2 bilobed, entire, shorter than L1, medial lobule larger; L3 slightly notched on lateral side or entire, bilobed but with outer lobule membranous, subequal or slightly smaller than inner lobule. **Gland spines.** Gland spine formula 1-1-1 (microduct formula 1-1-1), with 1 gland spine near each body margin of abdominal segment 5; without gland spines between L1. **Ducts.** Large macroducts in submedian area of segments 5 and 6 (with 4 – 7 (5) on segment 5 and 4 – 5 (5) on segment 6), in submarginal areas of segment 5 (with 5 – 7 (7)), and in marginal area of segments 5 to 7 (with 1 on segment 7, 2 on segment 6, 2 on segment 5); absent on segment 8. Largest macroduct on segment 7 (between L1 and L2) 15 – 20 (17.5) μm long. Small macroducts sparse on segment 5 (sometimes 2). Pygidial microducts on venter in submarginal areas of segment 5 to 7, with 0 – 2 (2) ducts on segment 5, 1 – 4 (2) ducts on segment 6 and 1 – 2 (2) ducts on segment 7; pygidial microducts absent from dorsum. **Pores.** Perivulvar pores with 5 loculi, in 5 groups, 1 median with 12 – 24 (15) pores, 2 anterolateral with 24 – 35 (28) pores, 2 posterolateral with 27 – 33 (30) pores. **Anal opening.** Located 7.7 – 16.3 (11) times length of anal opening from base of median lobes, diameter 10 – 17.5 (14.5) μm long. **Setae.** Dorsal setae: 2 setose on L1, 1 setose (~ 11 µm) between lobules of L2 and L3. Ventral setae: 1 small on L1, 1 marginal at base of each gland spine cluster and 1 on submarginal area of each segment, 2 in submedian area of segment 6, half as long as dorsal setae; 2 pairs of setae in a row anterior to the vulva.

**Prepygidium: Gland spines.** Near each body margin from segment 2 to 4, absent from mesothorax, metathorax and segment 1; 4 – 8 (6) on segment 2, 4 – 7 (5) on segment 3 and 1 – 4 (2) on segment 4. Gland spines on segments 3 and 4 protruding from margin and about same size as those on segment 5. Gland spines on segment 2 the smallest and never protruding from the margin. **Ducts**. Macroducts of 2 sizes; larger macroducts in submedian areas of abdominal segments 4 and 3. Small macroducts in submedian area of any or all of segments 3 and 4, in marginal areas from meso- or metathorax to segment 3. Prepygidial microducts almost absent on both surfaces, with a few on segment 2.

**Cephalothorax:** Microducts sparse on venter and dorsum, with a slight concentration around posterior thoracic spiracles and head. Perispiracular pores with 3 loculi, anterior spiracles with 5 – 6 (6) pores, posterior spiracles with 1 – 3 (2) pores. Eyes represented by small sclerotized area, located on body margin at level near anterior clypeolabral shield. Antennae each with 1 long seta and 2 minute setae, distance between antennae 60 – 117.5 (80) µm.

#### Etymology.

The epithet *sonorae* is a Latin noun, the genitive form of Sonora, meaning “of Sonora”.

### Chionaspis torreyanae

Vea sp. n.

urn:lsid:zoobank.org:act:1EF8C3A2-CE72-420E-BE4D-9B07AD27BBA5

http://species-id.net/wiki/Chionaspis_torreyanae

Figure 4

#### Morphogroup E in Gwiazdowski et al. (2011).

#### Type material.

**Type location:** U.S.A., California, Channel Islands, Santa Rosa Island, 33°59.09'N, 120°1.42'W, on needle of *Pinus torreyana insularis* Schoenherr *et al*., 23 January 2008, C. Greene coll.

#### Type specimen:

Holotype adult female, slide-mount in balsam. Original label “D2238A, USA: California, Santa Rosa Island, 23.i.2008, 33°59'5.4"N, 120°01'25.4"W, Carolyn Greene, *Pinus torreyana insularis* ”, deposited at UMAM.

Paratypes: Adult females on separate slides, D2238D, E and G, same information as D2238A, deposited at USNM.

Other material examined: Adult female, slide-mount in balsam, original label “D1557A, USA: California, San Diego, 30.viii.2006, 32°56'27.2"N, 117°15'41.0"W, Rodger Gwiazdowski, *Pinus torreyana*”, deposited at UMAM. Adult females on separate slides, D1557D, E, F and G, same collection as D1557A, deposited at UMAM.

Adult female, slide-mount in balsam, original label “D1559A, USA: California, San Diego, 30.iii.2006, 32°55'12.9"N, 117°15'09.9"W, R. Gwiazdowski, *Pinus torreyana*’, deposited at UMAM. Adult female, D1559C, same collection data as D1559A, deposited at UMAM.

Adult female, slide-mount in balsam, original label “D2235A, USA: California, Santa Rosa Island, 23.i.2008, 33°59'04"N, 120°01'34.9"N, Carolyn Greene, *Pinus torreyana insularis* ”, deposited at USNM. DNA:AMCC: 205821. Adult female, slide-mount in balsam, original label “D2236A, USA: California, Santa Rosa Island, 23.i.2008, 33°59’4.9"N, 120°01’35"W, Carolyn Greene, *Pinus torreyana insularis*”, deposited at UMAM.

Adult female, slide mount in balsam, original label “D2240A, USA: California, Santa Rosa Island, 33°59'2.3"N, 120° 1'11.7"W”, Carolyn Greene, *Pinus torreyana insularis*”, deposited at UMAM. Adult females on separate slides, D2240C and D, same collection data as D2240A, deposited at UMAM.

#### Diagnosis.

*Chionaspis torreyanae* Vea differs from other *Chionaspis* with the combination of following characters (Table 1): gland spine formula 2-2-2, microduct formula 2-2-2, other abdominal gland spines usually each subtended by 2 microducts, unnotched pygidial lobes.

#### Description.

**Field characters:** All pine-feeding *Chionaspis* reported here, including *Chionaspis heterophyllae* and *Chionaspis pinifoliae* are indistinguishable by eye in the field. See the description above for *Chionaspis brachycephalon* Vea.

**Figure 4.**
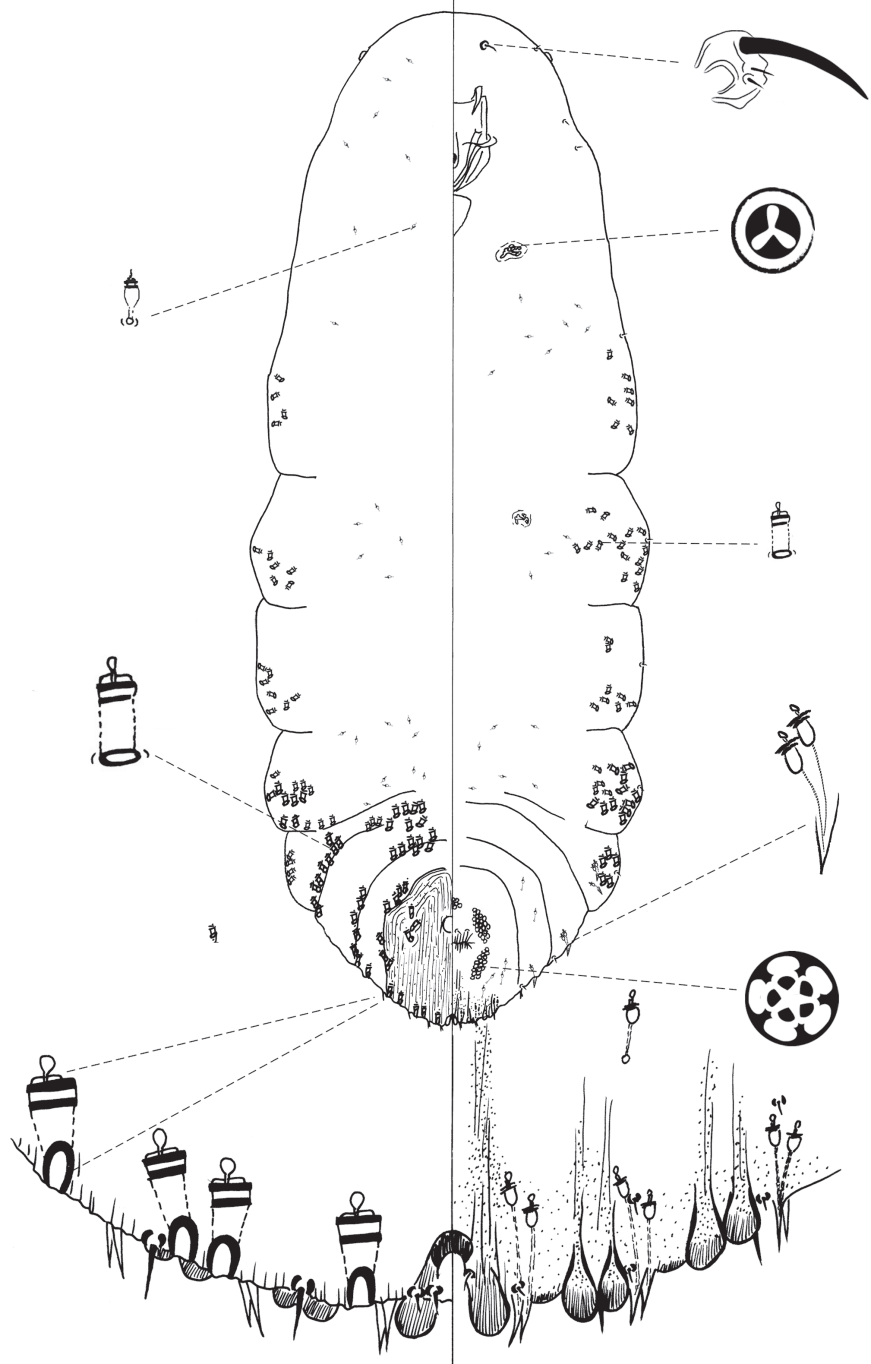
*Chionaspis torreyanae* Vea sp. n., adult female.

**Slide-mounted adult female** (Figure 4): spindle-shaped and elongate, lobed laterally and broader posteriorly (generally broadest at metathorax), length of holotype 1.55 mm, range (n=16) 1.15 mm – 1.65 mm; maximum width of holotype: 0.65 mm; range (n=16) 0.475 – 0.875 mm, maximum width at metathorax, rarely on first abdominal segment.

**Pygidium: Lobes**. Posterior margin with 3 pairs of definite lobes (L1, L2 and L3), fourth pair (L4) of lobes appear as series of low, sclerotized points; paraphyses absent. L1 separated by space 0.3 – 1 (0.6) times width of lobes, with a thick, protruding, U-shaped yoke uniting L1, lateral margins of lobes parallel, slightly diverging near apex, medial margin parallel-sided. L1 usually entire (1 minute notch may be present); L2 bilobed, smaller than L1, medial lobule always larger, both lobules entire; L3 bilobed, lateral lobule usually obsolete, or, when present, shorter than medial lobule but about equal in width. **Gland spines.** Gland spine formula 2-2-2 (microduct formula 2-2-2), with 1 short gland spine near each body margin on abdominal segment 5; without gland spines between median lobes. **Ducts.** Large macroducts in submedian area of segments 5 and 6 (with 4 – 6 (5) on segment 5 and 3 – 6 (4) on segment 6); in submarginal areas of segment 5 (with 5 – 8 (6)); in marginal area of segments 5 to 7 (with 2 – 3 (2) on segment 5, 2 on segment 6 and 1 on segment 7); absent on segment 8. Largest macroduct on segment 7 (between L1 and L2) 15 – 22.5 (20) μm long. Pygidial microducts always on venter in submarginal areas of segment 5 to 7, with 1 – 2 (1) duct on segment 5, 2 ducts on segment 6 and 1 duct on segment 7; pygidial microducts absent from dorsum. **Pores.** Perivulvar pores with 5 loculi, in 5 groups, 1 median with 8 – 17 (8) pores, 2 anterolateral with 20 – 27 (23) pores, 2 posterolateral with 17 – 26 (20) pores. **Anal opening.** Located 6.1 – 11.2 (9) times length of anal opening from base of median lobes, diameter 12.5 – 17.5 (15) μm. **Setae.** Dorsal setae: 2 setose on L1, 1 setose (~ 11 m) between lobules of L2 and L3. Ventral setae: 1 small on L1, 1 marginal at base of each gland spine cluster and 1 in submarginal area of each segment, 2 in submedian area of segment 6, small and short; 2 pairs of setae in a row anterior to the vulva.

**Prepygidium: Gland spines.** Near each body margin from segment 1 or 2 to 4, absent from mesothorax and metathorax; with 0 – 3 (0) on segment 1, 1 – 7 (3) on segment 2, 2 – 4 (3) on segment 3 and 1 – 2 (2) gland spine on segment 4 with 2 microducts extending, short and protruding from margin. Gland spines from segment 1 to 3 the smallest and never protruding from margin. **Ducts.** Macroducts of 2 sizes; largest macroducts in submedian and submarginal areas of abdominal segments 4 and 3. Small macroducts in submedian area of either or both of segments 3 and 4, in marginal areas from meso- or metathorax to segment 3. Prepygidial microducts sparsely present on venter and dorsum from segment 1 to 4.

**Cephalothorax:** Microducts sparsely present on venter and dorsum.Perispiracular pores primarily with 3 loculi, anterior spiracles with 5 – 8 (6) pores, posterior spiracles with 2 – 5 (3) pores. Eyes represented by small sclerotized area, located on body margin at level near anterior clypeolabral shield. Antennae each with 1 long seta and 2 shorter setae, distance between two antennae 65 – 135 (85) μm.

#### Etymology.

The epithet *torreyanae* is a Latin noun, genitive case, meaning “of *torreyana*”, referring to the pine species *Pinus torreyana*, on which *Chionaspis torreyanae* Vea was collected.

### Key to the species of pine feeding *Chionaspis* in North America:

**Table:** 

1	Gland spine present between median lobes; zygosis absent between medial lobes (Figure 2)	*Chionaspis caudata* Vea, sp. n.
–	Gland spine absent between median lobes; zygosis present between median lobes	2
2	Head reduced, with margins converging rapidly toward anterior end of cephalothorax; gland spine cluster in 1st, 2nd, and 3rd spaces each with 2 or more microducts (Figure 1)	*Chionaspis brachycephalon* Vea, sp. n.
–	Head normally developed, rounded; gland spine cluster in 1st, 2nd, and 3rd spaces each with 1 or more microducts	3
3	Gland spine cluster in 1^st^, 2^nd^ and 3^rd^ spaces each with a single microduct	4
–	Gland spine cluster in 1^st^, 2^nd^ and 3^rd^ spaces each with two microducts (Figure 4)	*Chionaspis torreyanae* Vea, sp. n.
4	Medial margins of median lobes curving abruptly outward near midpoint between base and apex: parallel near base and becoming suddenly divergent (and slightly notched) in apical half (Figure 3)	*Chionaspis sonorae* Vea, sp. n.
–	Medial margins of median lobes not curving abruptly near midpoint: either diverging throughout their length, or parallel for most of their length and slightly diverging near apex	5
5	Space between median lobes, at midpoint between base and apex, > 1.5 width of median lobe; median lobes usually continually diverging throughout their length	*Chionaspis heterophyllae* Cooley
–	Space between median lobes, at midpoint between base and apex, < 1.5 width of median lobe; median lobes usually parallel for much of their length	*Chionaspis pinifoliae* (Fitch)

## Discussion

### *Chionaspis caudata* Vea and a modified diagnosis of the genus *Chionaspi*s

The gland spine located between the median lobes of *Chionaspis caudata* Vea is unique among the species of *Chionaspis*. Takagi (1985), in his description of the genus, describes the median lobes as “united together in a basal zygosis”. If considering this character, conventional taxonomy would not place *Chionaspis caudata* Vea in *Chionaspis*, even though this species also possesses a suite of morphological features consistent with the genus, such as the relative position of perivulvar pores, macroducts and gland spines on the pygidium and other abdominal segments. Ferris’s (1937) key to North American diaspidids, still the most useful resource for the Mexican fauna, is utterly confounded by *Chionaspis caudata* Vea (it comes closest to the genus *Pseudoparlatoria* Cockerell, which has pair of conjoined gland spines between the median lobes). The phylogenetic results from Gwiazdowski et al. (2011) unambiguously place *Chionaspis caudata* Vea within the pine-feeding *Chionaspis* species complex, and the genus *Chionaspis*. Mexico is an undersampled region where specimens have only been collected recently, and these recent collections indicate that the genus *Chionaspis* is more variable than previously thought, especially regarding variation concerning key characters involving the pygidial median lobes.

### Morphogroup A and other yet-undescribed species

Gwiazdowski et al. (2011) mentioned a fifth novel morphogroup, Morphogroup A, which we have not described here. Although this group of specimens (from *Pinus cembroides* in the state of Queretaro) at first appeared to have a distinctive morphology, we found it challenging to write a key that could consistently discriminate it from *Chionaspis pinifoliae*, so we have conservatively omitted to describe it here. The molecular evidence of Gwiazdowski et al. (2011) suggests that several additional species of pine-feeding *Chionaspis* remain undescribed.

## Supplementary Material

XML Treatment for Chionaspis brachycephalon

XML Treatment for Chionaspis caudata

XML Treatment for Chionaspis sonorae

XML Treatment for Chionaspis torreyanae

## Appendix 1

Voucher information for all type specimens. (doi: 10.3897/zookeys.270.2910.app1) File format: Microsoft Word document (doc). **Explanation note:** Voucher information for all type specimens; each specimen has several kinds of vouchered material. Specimens are conventionally slidemounted in balsam, and each is associated with extracted, whole genomic DNA. Additionally type lots from which the specimens have been drawn are preserved as in-situ frozen tissue associated with host plant tissue, and some specimens have DNA sequence data vouchered in GenBank. The Sample ID number directly corresponds to Sample ID number in Appendix 2.

## Appendix 2

Locality information for all type specimens. (doi: 10.3897/zookeys.270.2910.app2) File format: Microsoft Word document (doc). **Explanation note:** Locality information for all type specimens; the Sample ID number directly corresponds to Sample ID number in Appendix 2. The geodetic system used for all GPS points is WGS 1984.

## References

[B1] Andersen JC Normark BB Morse GE Gruwell ME (2010) Cryptic diversity in the *Aspidiotus nerii* complex in Australia. Annals of the Entomological Society of America 103: 844-854. doi: 10.1603/AN10060

[B2] Andresen JW (1957)*Phenacaspis heterophyllae* Cooley in New Jersey. Journal of the New York Entomological Society 65: 81–84. link: http://www.jstor.org/stable/10.2307/25005619

[B3] Balachowsky AS (1930) Deux *Chionaspis* (Hem. Coccidae) nouveaux de l’*Abies pinsapo* et du cèdre. Bulletin de la Société Entomologique de France 17: 266–273.

[B4] Ben-Dov Y Miller DR Gibson GAP (2012) ScaleNet, Scales in a Family Query Results. 25 November 2012. http://www.sel.barc.usda.gov/scalecgi/chklist.exe?Family=Diaspididae&genus=

[B5] Benson DA Karsch-Mizrachi I Lipman DJ Ostell J Sayers EW (2012) Genbank. Nucleic Acids Research 40: D48–53. doi: 10.1093/nar/gkr1202 PMC3245039 22144687

[B6] Bickford D Lohman DJ Sodhi NS Ng PKL Meier R Winker K Ingram KK Das I (2007) Cryptic species as a window on diversity and conservation. Trends Ecology and Evolution 22: 148-155. doi: 10.1016/j.tree.2006.11.004 17129636

[B7] Borchsenius NS (1966) A catalogue of the armoured scale insects (Diaspidoidea) of the world. Nauka, Moscow & Leningrad, 449 pp.[In Russian]

[B8] Comstock JH (1881) Report of the Entomologist. Report of the Commissioner of Agriculture, United States Department of Agriculture 1880/1881: 276–349.

[B9] Cook LG Edwards RD Crisp MD and Hardy NB (2010) Need morphology always be required for new species descriptions? Invertebrate Systematics 24: 322–326. doi: 10.1071/IS10011

[B10] Cooley RA (1897) New species of *Chionaspis*. Canadian Entomologist 29: 278-282.

[B11] Cooley RA (1899) The coccid genera *Chionaspis* and *Hemichionaspis*. Special Bulletin, Hatch Experiment Station of the Massachusetts Agricultural College 1899: 1-57.

[B12] Dones RA Evans GA (2011) A new species of armored scale, *Mycetaspis ailynaomi* (Hemiptera, Diaspididae, Aspidiotinae), associated with *Mammea americana* L. (Malpighiales, Calophyllaceae) from Puerto Rico. ZooKeys 108: 1-10. doi:** **10.3897/zookeys.108.1214 10.3897/zookeys.108.1214 PMC3119312 21852924

[B13] Evans GA Watson GW Miller DR (2009) A new species of armored scale (Hemiptera: Coccoidea: Diaspididae) found on avocado fruit from Mexico and a key to the species of armored scales found on avocado worldwide. Zootaxa 1991: 57–68. link: http://www.sel.barc.usda.gov/Coccoidea/Evans.pdf

[B14] Ferris GF (1937) Atlas of the scale insects of North America. Series I. Stanford University Press, Stanford, CA.

[B15] Fitch A (1856) The pine-leaf scale insect, *Aspidiotus pinifoliae*, new species. In Second report on noxious, beneficial, and other insects of the state of New York. Transactions of the New York State Agricultural Society with an Abstract of the Proceedings of the County Agricultural Societies 15: 488-494.

[B16] García Mercet R (1912) Los enemigos parásitos de las plantas: Los afelininos. Trabajos del Museo de Ciencias Naturales no. 10, Imprenta de Eduardo Arias, Madrid, 306 pp.

[B17] Gwiazdowski RA Vea IM Andersen J Normark BB (2011) Discovery of cryptic species among North American pine-feeding *Chionaspis* scale insects (Hemiptera: Diaspididae). Biological Journal of the Linnean Society 47: 47-62. doi: 10.1111/j.1095-8312.2011.01716.x

[B18] Gwiazdowski RA (2011) Discovery of cryptic species diversity in North American Pine-feeding *Chionaspis* scale insects (Hemiptera: Diaspididae) Organismic and Evolutionary Biology & Entomology. PhD thesis, University of Massachusetts, Amherst, Amherst.

[B19] Hudson RR Coyne JA (2002) Mathematical consequences of the genealogical species concept. Evolution 56: 1557–1565. link: http://www.ncbi.nlm.nih.gov/pubmed/12353748 10.1111/j.0014-3820.2002.tb01467.x 12353748

[B20] LeBaron W (1872) The willow bark-louse (*Mytilaspis salicis* n. sp.) Annual Report, Noxious Insects of the State of Illinois 2: 140.

[B21] Lindinger L (1935) Die nunmehr gültigen Namen der Arten in meinem ‘Schildläusebuch’ und in den ‘Schildläusen der Mitteleuropäischen Gewächshäuser’. Entomologisches Jahrbuch 44: 127-149.

[B22] Liu T-X Rhoades M Bullington SW Kosztarab M Jiang G-Z (1989) Biosystematics of the adult females of the genus Chionaspis (Homoptera: Coccoidea: Diaspididae) of North America, with emphasis on polymorphism. Virginia Agricultural Experiment Station, Virginia Polytechnic Institute and State University, Blacksburg, VA, 198 pp.

[B23] MacGillivray AD (1921) The Coccidae. Tables for the Identification of the Subfamilies and Some of the More Important Genera and Species, together with Discussions of their Anatomy and Life History. Scarab, Urbana, IL, 520 pp.

[B24] Miller DR (1996) Checklist of the scale insects (Coccoidea: Homoptera) of Mexico. Proceedings of the Entomological Society of Washington 98: 68-86. link: http://www.sel.barc.usda.gov/Coccoidea/Mexico.pdf

[B25] Miller DR Davidson JA (2005) Armored Scale Insect Pests of Trees and Shrubs (Hemiptera : Diaspididae). Cornell University Press, Ithaca, NY, 456 pp.

[B26] Neigel JE Avise J (1986) Phylogenetic relationships of mitochondrial DNA under various demographic models of speciation. In: Karlin S Nevo E (Eds). Evolutionary processes and theory. Academic Press, Orlando, FL: 515-534.

[B27] Nielsen DG (1970) Host impact, population dynamics, and chemical control of the pine needle scale, Phenacaspis pinifoliae (Fitch), in central New York. PhD Thesis, Cornell University, Ithaca.

[B28] Rugman-Jones PF Morse JG Stouthamer R (2009) Rapid Molecular Identification of Armored Scale Insects (Hemiptera: Diaspididae) on Mexican ‘Hass’ Avocado. Journal of Economic Entomology 102: 1948-1953. doi: 10.1603/029.102.0527 19886461

[B29] Shour MH (1986) Life History studies of the Pine Scale *Chionaspis heterophyllae* Cooley and the Pine Needle Scale, *C. pinifoliae* (Fitch). PhD Dissertation, Purdue University.

[B30] Takagi S (1985) The scale insect genus *Chionaspis*: a revised concept (Homoptera: Coccoidea: Diaspididae). Insecta matsumurana. Series entomology. New series, 33, 1–77. Avalible at http://eprints.lib.hokudai.ac.jp/dspace/handle/2115/9832

[B31] Veilleux K Miller DR Ben-Dov Y (2011) ScaleNet. *Chionaspis heterophyllae*: http://www.sel.barc.usda.gov/scalecgi/refsfor.exe?Family=Diaspididae&genus=Chionaspis&species=heterophyllae&subspecies=&begdate=&enddate=;*Chionaspis pinifoliae*: http://www.sel.barc.usda.gov/scalecgi/refsfor.exe?Family=Diaspididae&genus=Chionaspis&species=pinifoliae&subspecies=&begdate=&enddate= [27 November 2012]

[B32] Watson GW (2005) Diaspididae of the World, Arthropods of Economic Importance. ETI Bioinformatics: World Biodiversity Database http://wbd.etibioinformatics.nl/bis/diaspididae.php?menuentry=soorten&id=90

[B33] Wolff VRS Claps LE (2010) About *Diaspidistis* (Hemiptera, Diaspididae) with description of two new species. Iheringia Série Zoologia 100: 225-232. doi: 10.1590/S0073-47212010000300007

