# Supplementary material for: Corroborating molecular species discovery: Four new pine-feeding species of Chionaspis (Hemiptera, Diaspididae)
Source: Zookeys. 2013 Feb 18;(270):37–58. doi: 10.3897/zookeys.270.2910 (PMC3652468; doi:10.3897/zookeys.270.2910)
Supplement: Supplementary file 5 — Voucher information for all type specimens. (doi: 10.3897/zookeys.270.2910.app1) File format: Microsoft Word document (doc). Explanation note: Voucher information for all type specimens; each specimen has several kinds of vouchered material. Specimens are conventionally slidemounted in balsam, and each is associated with extracted, whole genomic DNA. Additionally type lots from which the specimens have been drawn are preserved as in-situ frozen tissue associated with host plant tissue, and some specimens have DNA sequence data vouchered in GenBank. The Sample ID number directly corresponds to Sample ID number in Appendix 2. [file ZooKeys-270-037-s001.doc]

Table 2a.

|  |  |  |  | **Slide mounts** | | **DNA catalog number** | | **Type lots with in-situ specimens** | |  | | |
| --- | --- | --- | --- | --- | --- | --- | --- | --- | --- | --- | --- | --- |
| **Genus** | **Species** | **Sample ID** | **Type** | **Slide repository** | **UMAM barcode** | **AMCC** | **UMAM** | **AMCC** | **UMAM** | **Genbank 28s** | **Genbank Ef1a** | **Genbank CO1-CO2** |
| *Chionaspis* | *brachycephalon* | D1765A | Holotype | CNIN | UMAM_ENT 00004297 | 205806 | - |  | - | GU349373 | GU350121 | GU349747 |
| *Chionaspis* | *brachycephalon* | D1765 | Type lot | - | - |  |  | 160916 | D1765 | - | - | - |
| *Chionaspis* | *brachycephalon* | D1765B | Paratype | NMNH | UMAM_ENT 00004298 | 205807 | - |  | - | - | - | - |
| *Chionaspis* | *brachycephalon* | D1718 | Type lot | - | - |  |  | 160867 | D1718 | - | - | - |
| *Chionaspis* | *brachycephalon* | D1718A | Paratype | UMAM | UMAM_ENT 00004293 | 205808 | - |  | - | GU349342 | GU350090 | GU349716 |
| *Chionaspis* | *brachycephalon* | D1718C | Paratype | UMAM | UMAM_ENT 00004294 | - | D1718C | - |  | - | - | - |
| *Chionaspis* | *brachycephalon* | D1718D | Paratype | UMAM | UMAM_ENT 00004295 | - | D1718D | - |  | - | - | - |
| *Chionaspis* | *brachycephalon* | D1718F | Paratype | UMAM | UMAM_ENT 00004296 | - | D1718F | - |  | - | - | - |
| *Chionaspis* | *caudata* | D1703D | Holotype | CNIN | UMAM_ENT 00004280 | 205809 | - |  | - | - | - | - |
| *Chionaspis* | *caudata* | D1703 | Type Lot | - | - |  |  | 160852 | D1703 |  |  |  |
| *Chionaspis* | *caudata* | D1703A | Paratype | NMNH | UMAM_ENT 00004284 | 205810 | - |  | - | GU349330 | GU350078 | GU349704 |
| *Chionaspis* | *caudata* | D1703F | Paratype | UMAM | UMAM_ENT 00004286 | 205812 | - |  | - | - | - | - |
| *Chionaspis* | *caudata* | D1703G | Paratype | NMNH | UMAM_ENT 00004287 | 205811 | D1703G |  |  | - | - | - |
| *Chionaspis* | *caudata* | D1703H | Paratype | UMAM | UMAM_ENT 00004288 | - | D1703H | - |  | - | - | - |
| *Chionaspis* | *caudata* | D1703I | Paratype | UMAM | UMAM_ENT 00004289 | - | D1703I | - |  | - | - | - |
| *Chionaspis* | *caudata* | D1703J | Paratype | UMAM | UMAM_ENT 00004290 | - | D1703J | - |  | - | - | - |
| *Chionaspis* | *caudata* | D1703K | Paratype | UMAM | UMAM_ENT 00004291 | - | D1703K | - |  | - | - | - |
| *Chionaspis* | *caudata* | D1703L | Paratype | UMAM | UMAM_ENT 00004292 | - | D1703L | - |  | - | - | - |
| *Chionaspis* | *caudata* | D1702A | Paratype | UMAM | UMAM_ENT 00004283 | 205813 | - |  | - | - | - | - |
| *Chionaspis* | *caudata* | D1702 | Type Lot | - | - |  |  | 160851 | D1702 | - | - | - |
| *Chionaspis* | *sonorae* | D1781A | Holotype | CNIN | UMAM_ENT 00004307 | 205816 | - |  | - | GU349387 | GU350135 | GU349761 |
| *Chionaspis* | *sonorae* | D1781 | Type Lot | - | - |  |  | 160933 | D1781 | - | - | - |
| *Chionaspis* | *sonorae* | D1781C | Paratype | UMAM | UMAM_ENT 00000724 | - | D1781C | - |  | - | - | - |
| *Chionaspis* | *sonorae* | D1781F | Paratype | UMAM | UMAM_ENT 00004308 | - | D1781F | - |  | - | - | - |
| *Chionaspis* | *sonorae* | D1780A | Paratype | USNM | UMAM_ENT 00004299 | 205817 | - |  | - | GU349386 | GU350134 | GU349760 |
| *Chionaspis* | *sonorae* | D1780 | Type lot | - | - |  |  | 160932 | D1780 | - | - | - |
| *Chionaspis* | *sonorae* | D1780B | Paratype | UMAM | UMAM_ENT 00004300 | 205818 | - |  | - | - | - | - |
| *Chionaspis* | *sonorae* | D1780C | Paratype | UMAM | UMAM_ENT 00000722 | - | D1780C | - |  | - | - | - |
| *Chionaspis* | *sonorae* | D1780D | Paratype | UMAM | UMAM_ENT 00004301 | - | D1780D | - |  | - | - | - |
| *Chionaspis* | *sonorae* | D1780E | Paratype | UMAM | UMAM_ENT 00004302 | - | D1780E | - |  | - | - | - |
| *Chionaspis* | *sonorae* | D1780F | Paratype | UMAM | UMAM_ENT 00004303 | - | D1780F | - |  | - | - | - |
| *Chionaspis* | *sonorae* | D1780G | Paratype | UMAM | UMAM_ENT 00004306 | - | D1780G | - |  | - | - | - |
| *Chionaspis* | *torreyanae* | D2238A | Holotype | UMAM | UMAM_ENT 00004311 | 205819 | - |  | - | GU349431 | GU350178 | GU349805 |
| *Chionaspis* | *torreyanae* | D2238 | Type Lot | - | - |  |  | 167736 | D2238 | - | - | - |
| *Chionaspis* | *torreyanae* | D2238D | Paratype | UMAM | UMAM_ENT 00004312 | - | D2238D | - |  | - | - | - |
| *Chionaspis* | *torreyanae* | D2238E | Paratype | UMAM | UMAM_ENT 00004313 | - | D2238E | - |  | - | - | - |
| *Chionaspis* | *torreyanae* | D2238G | Paratype | UMAM | UMAM_ENT 00004314 | - | D2238G | - |  | - | - | - |
| *Chionaspis* | *torreyanae* | D1557A | Paratype | UMAM | UMAM_ENT 00004305 | 205822 | - |  | - | GU349190 | GU349938 | GU349564 |
| *Chionaspis* | *torreyanae* | D1557 | Type Lot | - | - |  |  | 160706 | D1557 | - | - | - |
| *Chionaspis* | *torreyanae* | D1557D | Paratype | UMAM | UMAM_ENT 00004278 | - | D1557D | - |  | - | - | - |
| *Chionaspis* | *torreyanae* | D1557E | Paratype | UMAM | UMAM_ENT 00004279 | - | D1557E | - |  | - | - | - |
| *Chionaspis* | *torreyanae* | D1557F | Paratype | UMAM | UMAM_ENT 00004280 | - | D1557F | - |  | - | - | - |
| *Chionaspis* | *torreyanae* | D1557G | Paratype | UMAM | UMAM_ENT 00004281 | - | D1557G | - |  | - | - | - |
| *Chionaspis* | *torreyanae* | D1559A | Paratype | UMAM | UMAM_ENT 00004282 | 205824 | - |  | - | - | - | - |
| *Chionaspis* | *torreyanae* | D1559 | Type Lot | - | - |  |  | 160708 | D1559 | - | - | - |
| *Chionaspis* | *torreyanae* | D1559C | Paratype | UMAM | UMAM_ENT 00000676 | - | D1559C | - |  | - | - | - |
| *Chionaspis* | *torreyanae* | D2235A | Paratype | NMNH | UMAM_ENT 00004309 | 205821 | - |  | - | GU349429 | GU350176 | GU349803 |
| *Chionaspis* | *torreyanae* | D2235 | Type Lot | - | - |  |  | 167733 | D2235 | - | - | - |
| *Chionaspis* | *torreyanae* | D2236A | Paratype | UMAM | UMAM_ENT 00004310 | 205820 | - |  | - | GU349430 | GU350177 | GU349804 |
| *Chionaspis* | *torreyanae* | D2236 | Type Lot | - | - |  |  | 167734 | D2236 | - | - | - |
| *Chionaspis* | *torreyanae* | D2240A | Paratype | UMAM | UMAM_ENT 00000949 | - | D2240A | - |  | - | - | - |
| *Chionaspis* | *torreyanae* | D2240 | Type Lot | - | - |  |  | 167738 | D2240 | - | - | - |
| *Chionaspis* | *torreyanae* | D2240C | Paratype | UMAM | UMAM_ENT 00004315 | - | D2240C | - |  | - | - | - |
| *Chionaspis* | *torreyanae* | D2240D | Paratype | UMAM | UMAM_ENT 00004316 | - | D2240D | - |  | - | - | - |
