# Supplementary material for: Corroborating molecular species discovery: Four new pine-feeding species of Chionaspis (Hemiptera, Diaspididae)
Source: Zookeys. 2013 Feb 18;(270):37–58. doi: 10.3897/zookeys.270.2910 (PMC3652468; doi:10.3897/zookeys.270.2910)
Supplement: Supplementary file 6 — Locality information for all type specimens. (doi: 10.3897/zookeys.270.2910.app2) File format: Microsoft Word document (doc). Explanation note: Locality information for all type specimens; the Sample ID number directly corresponds to Sample ID number in Appendix 2. The geodetic system used for all GPS points is WGS 1984. [file ZooKeys-270-037-s002.doc]

Table 2b.

| **Genus** | **Species** | **Sample ID** | **Type** | **Country** | **State** | **Locality** | **Host Genus** | **HostSpecies** | **Collector** | **Coll. date** | **Lat/Long hddd.ddddd** | **Lat/Long hddd mm ss.s** | **Elev. M** |
| --- | --- | --- | --- | --- | --- | --- | --- | --- | --- | --- | --- | --- | --- |
| *Chionaspis* | *brachycephalon* | D1765A | Holotype | Mexico | Durango | Resturante “Los Pinos”, Navios | *Pinus* | *cooperi* | R. Gwiazdowski, A. G. Arévalo | 24-Sep-2007 | N23.89913 W105.04710 | N23 53 56.9 W105 02 49.6 | 2487 |
| *Chionaspis* | *brachycephalon* | D1765 | Type lot | Mexico | Durango | Resturante “Los Pinos”, Navios | *Pinus* | *cooperi* | R. Gwiazdowski, A. G. Arévalo | 24-Sep-2007 | N23.89913 W105.04710 | N23 53 56.9 W105 02 49.6 | 2487 |
| *Chionaspis* | *brachycephalon* | D1765B | Paratype | Mexico | Durango | Resturante “Los Pinos”, Navios | *Pinus* | *cooperi* | R. Gwiazdowski, A. G. Arévalo | 24-Sep-2007 | N23.89913 W105.04710 | N23 53 56.9 W105 02 49.6 | 2487 |
| *Chionaspis* | *brachycephalon* | D1718 | Type lot | Mexico | Mexico | Hwy 95 South of Tres Marias | *Pinus* | *pseudostrobus* | R. Gwiazdowski, D. Gernandt | 1-Sep-2007 | N19.02709 W99.20977 | N19 01 37.5 W99 12 35.2 | 2579 |
| *Chionaspis* | *brachycephalon* | D1718A | Paratype | Mexico | Mexico | Hwy 95 South of Tres Marias | *Pinus* | *pseudostrobus* | R. Gwiazdowski, D. Gernandt | 1-Sep-2007 | N19.02709 W99.20977 | N19 01 37.5 W99 12 35.2 | 2579 |
| *Chionaspis* | *brachycephalon* | D1718C | Paratype | Mexico | Mexico | Hwy 95 South of Tres Marias | *Pinus* | *pseudostrobus* | R. Gwiazdowski, D. Gernandt | 1-Sep-2007 | N19.02709 W99.20977 | N19 01 37.5 W99 12 35.2 | 2579 |
| *Chionaspis* | *brachycephalon* | D1718D | Paratype | Mexico | Mexico | Hwy 95 South of Tres Marias | *Pinus* | *pseudostrobus* | R. Gwiazdowski, D. Gernandt | 1-Sep-2007 | N19.02709 W99.20977 | N19 01 37.5 W99 12 35.2 | 2579 |
| *Chionaspis* | *brachycephalon* | D1718F | Paratype | Mexico | Mexico | Hwy 95 South of Tres Marias | *Pinus* | *pseudostrobus* | R. Gwiazdowski, D. Gernandt | 1-Sep-2007 | N19.02709 W99.20977 | N19 01 37.5 W99 12 35.2 | 2579 |
| *Chionaspis* | *caudata* | D1703D | Holotype | Mexico | Oaxaca | Oaxaca, Hwy 175 | *Pinus* | *patula_longipedunculata* | R. Gwiazdowski, M. Dahlberg | 28-Aug-2007 | N16.19979 W96.52526 | N16 11 59.3 W96 31 30.9 | 2381 |
| *Chionaspis* | *caudata* | D1703 | Type Lot | Mexico | Oaxaca | Oaxaca, Hwy 175 | *Pinus* | *patula_longipedunculata* | R. Gwiazdowski, M. Dahlberg | 28-Aug-2007 | N16.19979 W96.52526 | N16 11 59.3 W96 31 30.9 | 2381 |
| *Chionaspis* | *caudata* | D1703A | Paratype | Mexico | Oaxaca | Oaxaca, Hwy 175 | *Pinus* | *patula_longipedunculata* | R. Gwiazdowski, M. Dahlberg | 28-Aug-2007 | N16.19979 W96.52526 | N16 11 59.3 W96 31 30.9 | 2381 |
| *Chionaspis* | *caudata* | D1703F | Paratype | Mexico | Oaxaca | Oaxaca, Hwy 175 | *Pinus* | *patula_longipedunculata* | R. Gwiazdowski, M. Dahlberg | 28-Aug-2007 | N16.19979 W96.52526 | N16 11 59.3 W96 31 30.9 | 2381 |
| *Chionaspis* | *caudata* | D1703G | Paratype | Mexico | Oaxaca | Oaxaca, Hwy 175 | *Pinus* | *patula_longipedunculata* | R. Gwiazdowski, M. Dahlberg | 28-Aug-2007 | N16.19979 W96.52526 | N16 11 59.3 W96 31 30.9 | 2381 |
| *Chionaspis* | *caudata* | D1703H | Paratype | Mexico | Oaxaca | Oaxaca, Hwy 175 | *Pinus* | *patula_longipedunculata* | R. Gwiazdowski, M. Dahlberg | 28-Aug-2007 | N16.19979 W96.52526 | N16 11 59.3 W96 31 30.9 | 2381 |
| *Chionaspis* | *caudata* | D1703I | Paratype | Mexico | Oaxaca | Oaxaca, Hwy 175 | *Pinus* | *patula_longipedunculata* | R. Gwiazdowski, M. Dahlberg | 28-Aug-2007 | N16.19979 W96.52526 | N16 11 59.3 W96 31 30.9 | 2381 |
| *Chionaspis* | *caudata* | D1703J | Paratype | Mexico | Oaxaca | Oaxaca, Hwy 175 | *Pinus* | *patula_longipedunculata* | R. Gwiazdowski, M. Dahlberg | 28-Aug-2007 | N16.19979 W96.52526 | N16 11 59.3 W96 31 30.9 | 2381 |
| *Chionaspis* | *caudata* | D1703K | Paratype | Mexico | Oaxaca | Oaxaca, Hwy 175 | *Pinus* | *patula_longipedunculata* | R. Gwiazdowski, M. Dahlberg | 28-Aug-2007 | N16.19979 W96.52526 | N16 11 59.3 W96 31 30.9 | 2381 |
| *Chionaspis* | *caudata* | D1703L | Paratype | Mexico | Oaxaca | Oaxaca, Hwy 175 | *Pinus* | *patula_longipedunculata* | R. Gwiazdowski, M. Dahlberg | 28-Aug-2007 | N16.19979 W96.52526 | N16 11 59.3 W96 31 30.9 | 2381 |
| *Chionaspis* | *caudata* | D1702A | Paratype | Mexico | Oaxaca | Oaxaca, Hwy 175 | *Pinus* | *pseudostrobus_oaxacana* | R. Gwiazdowski, M. Dahlberg | 28-Aug-2007 | N16.17536 W96.50673 | N16 10 31.3 W96 30 24.2 | 2471 |
| *Chionaspis* | *caudata* | D1702 | Type Lot | Mexico | Oaxaca | Oaxaca, Hwy 175 | *Pinus* | *pseudostrobus_oaxacana* | R. Gwiazdowski, M. Dahlberg | 28-Aug-2007 | N16.17536 W96.50673 | N16 10 31.3 W96 30 24.2 | 2471 |
| *Chionaspis* | *sonorae* | D1781A | Holotype | Mexico | Sonora | Yecora | *Pinus* | *engelmannii* | R. Gwiazdowski T. R. Van Devender, A. L. Reina-G | 8-Oct-2007 | N28.37411 W108.93507 | N28 22 26.8 W108 56 06.3 | 1528 |
| *Chionaspis* | *sonorae* | D1781 | Type Lot | Mexico | Sonora | Yecora | *Pinus* | *engelmannii* | R. Gwiazdowski T. R. Van Devender, A. L. Reina-G | 8-Oct-2007 | N28.37411 W108.93507 | N28 22 26.8 W108 56 06.3 | 1528 |
| *Chionaspis* | *sonorae* | D1781C | Paratype | Mexico | Sonora | Yecora | *Pinus* | *engelmannii* | R. Gwiazdowski T. R. Van Devender, A. L. Reina-G | 8-Oct-2007 | N28.37411 W108.93507 | N28 22 26.8 W108 56 06.3 | 1528 |
| *Chionaspis* | *sonorae* | D1781F | Paratype | Mexico | Sonora | Yecora | *Pinus* | *engelmannii* | R. Gwiazdowski T. R. Van Devender, A. L. Reina-G | 8-Oct-2007 | N28.37411 W108.93507 | N28 22 26.8 W108 56 06.3 | 1528 |
| *Chionaspis* | *sonorae* | D1780A | Paratype | Mexico | Sonora | West of Yecora | *Pinus* | *engelmannii* | R. Gwiazdowski T. R. Van Devender, A. L. Reina-G | 7-Oct-2007 | N28.35931 W109.03007 | N28 21 33.5 W109 01 48.3 | 2106 |
| *Chionaspis* | *sonorae* | D1780 | Type lot | Mexico | Sonora | West of Yecora | *Pinus* | *engelmannii* | R. Gwiazdowski T. R. Van Devender, A. L. Reina-G | 7-Oct-2007 | N28.35931 W109.03007 | N28 21 33.5 W109 01 48.3 | 2106 |
| *Chionaspis* | *sonorae* | D1780B | Paratype | Mexico | Sonora | West of Yecora | *Pinus* | *engelmannii* | R. Gwiazdowski T. R. Van Devender, A. L. Reina-G | 7-Oct-2007 | N28.35931 W109.03007 | N28 21 33.5 W109 01 48.3 | 2106 |
| *Chionaspis* | *sonorae* | D1780C | Paratype | Mexico | Sonora | West of Yecora | *Pinus* | *engelmannii* | R. Gwiazdowski T. R. Van Devender, A. L. Reina-G | 7-Oct-2007 | N28.35931 W109.03007 | N28 21 33.5 W109 01 48.3 | 2106 |
| *Chionaspis* | *sonorae* | D1780D | Paratype | Mexico | Sonora | West of Yecora | *Pinus* | *engelmannii* | R. Gwiazdowski T. R. Van Devender, A. L. Reina-G | 7-Oct-2007 | N28.35931 W109.03007 | N28 21 33.5 W109 01 48.3 | 2106 |
| *Chionaspis* | *sonorae* | D1780E | Paratype | Mexico | Sonora | West of Yecora | *Pinus* | *engelmannii* | R. Gwiazdowski T. R. Van Devender, A. L. Reina-G | 7-Oct-2007 | N28.35931 W109.03007 | N28 21 33.5 W109 01 48.3 | 2106 |
| *Chionaspis* | *sonorae* | D1780F | Paratype | Mexico | Sonora | West of Yecora | *Pinus* | *engelmannii* | R. Gwiazdowski T. R. Van Devender, A. L. Reina-G | 7-Oct-2007 | N28.35931 W109.03007 | N28 21 33.5 W109 01 48.3 | 2106 |
| *Chionaspis* | *sonorae* | D1780G | Paratype | Mexico | Sonora | West of Yecora | *Pinus* | *engelmannii* | R. Gwiazdowski T. R. Van Devender, A. L. Reina-G | 7-Oct-2007 | N28.35931 W109.03007 | N28 21 33.5 W109 01 48.3 | 2106 |
| *Chionaspis* | *torreyanae* | D2238A | Holotype | USA | California | Santa Rosa Island | *Pinus* | *torreyana_var._insularis* | C. Greene | 23-Jan-2008 | N33.98483 W120.02373 | N33 59 5.4 W120 01 25.4 | 25 |
| *Chionaspis* | *torreyanae* | D2238 | Type Lot | USA | California | Santa Rosa Island | *Pinus* | *torreyana_var._insularis* | C. Greene | 23-Jan-2008 | N33.98483 W120.02373 | N33 59 5.4 W120 01 25.4 | 25 |
| *Chionaspis* | *torreyanae* | D2238D | Paratype | USA | California | Santa Rosa Island | *Pinus* | *torreyana_var._insularis* | C. Greene | 23-Jan-2008 | N33.98483 W120.02373 | N33 59 5.4 W120 01 25.4 | 25 |
| *Chionaspis* | *torreyanae* | D2238E | Paratype | USA | California | Santa Rosa Island | *Pinus* | *torreyana_var._insularis* | C. Greene | 23-Jan-2008 | N33.98483 W120.02373 | N33 59 5.4 W120 01 25.4 | 25 |
| *Chionaspis* | *torreyanae* | D2238G | Paratype | USA | California | Santa Rosa Island | *Pinus* | *torreyana_var._insularis* | C. Greene | 23-Jan-2008 | N33.98483 W120.02373 | N33 59 5.4 W120 01 25.4 | 25 |
| *Chionaspis* | *torreyanae* | D1557A | Paratype | USA | California | San Diego | *Pinus* | *torreyana* | R. Gwiazdowski | 30-Aug-2006 | N32.94089 W117.26139 | N32 56 27.2 W117 15 41.0 | 32 |
| *Chionaspis* | *torreyanae* | D1557 | Type Lot | USA | California | San Diego | *Pinus* | *torreyana* | R. Gwiazdowski | 30-Aug-2006 | N32.94089 W117.26139 | N32 56 27.2 W117 15 41.0 | 32 |
| *Chionaspis* | *torreyanae* | D1557D | Paratype | USA | California | San Diego | *Pinus* | *torreyana* | R. Gwiazdowski | 30-Aug-2006 | N32.94089 W117.26139 | N32 56 27.2 W117 15 41.0 | 32 |
| *Chionaspis* | *torreyanae* | D1557E | Paratype | USA | California | San Diego | *Pinus* | *torreyana* | R. Gwiazdowski | 30-Aug-2006 | N32.94089 W117.26139 | N32 56 27.2 W117 15 41.0 | 32 |
| *Chionaspis* | *torreyanae* | D1557F | Paratype | USA | California | San Diego | *Pinus* | *torreyana* | R. Gwiazdowski | 30-Aug-2006 | N32.94089 W117.26139 | N32 56 27.2 W117 15 41.0 | 32 |
| *Chionaspis* | *torreyanae* | D1557G | Paratype | USA | California | San Diego | *Pinus* | *torreyana* | R. Gwiazdowski | 30-Aug-2006 | N32.94089 W117.26139 | N32 56 27.2 W117 15 41.0 | 32 |
| *Chionaspis* | *torreyanae* | D1559A | Paratype | USA | California | San Diego | *Pinus* | *torreyana* | R. Gwiazdowski | 30-Aug-2006 | N32.92025 W117.25276 | N32 55 12.9 W117 15 09.9 | 94 |
| *Chionaspis* | *torreyanae* | D1559 | Type Lot | USA | California | San Diego | *Pinus* | *torreyana* | R. Gwiazdowski | 30-Aug-2006 | N32.92025 W117.25276 | N32 55 12.9 W117 15 09.9 | 94 |
| *Chionaspis* | *torreyanae* | D1559C | Paratype | USA | California | San Diego | *Pinus* | *torreyana* | R. Gwiazdowski | 30-Aug-2006 | N32.92025 W117.25276 | N32 55 12.9 W117 15 09.9 | 94 |
| *Chionaspis* | *torreyanae* | D2235A | Paratype | USA | California | Santa Rosa Island | *Pinus* | *torreyana_var._insularis* | C. Greene | 23-Jan-2008 | N33.98445 W120.02635 | N33 59 04 W120 01 34.9 | 62 |
| *Chionaspis* | *torreyanae* | D2235 | Type Lot | USA | California | Santa Rosa Island | *Pinus* | *torreyana_var._insularis* | C. Greene | 23-Jan-2008 | N33.98445 W120.02635 | N33 59 04 W120 01 34.9 | 62 |
| *Chionaspis* | *torreyanae* | D2236A | Paratype | USA | California | Santa Rosa Island | *Pinus* | *torreyana_var._insularis* | C. Greene | 23-Jan-2008 | N33.98468 W120.02638 | N33 59 4.9 W120 01 35 | 53 |
| *Chionaspis* | *torreyanae* | D2236 | Type Lot | USA | California | Santa Rosa Island | *Pinus* | *torreyana_var._insularis* | C. Greene | 23-Jan-2008 | N33.98468 W120.02638 | N33 59 4.9 W120 01 35 | 53 |
| *Chionaspis* | *torreyanae* | D2240A | Paratype | USA | California | Santa Rosa Island | *Pinus* | *torreyana_var._insularis* | C. Greene | 23-Jan-2008 | N33.98398 W120.01992 | N33 59 2.3 W120 01 11.7 | 28 |
| *Chionaspis* | *torreyanae* | D2240 | Type Lot | USA | California | Santa Rosa Island | *Pinus* | *torreyana_var._insularis* | C. Greene | 23-Jan-2008 | N33.98398 W120.01992 | N33 59 2.3 W120 01 11.7 | 28 |
| *Chionaspis* | *torreyanae* | D2240C | Paratype | USA | California | Santa Rosa Island | *Pinus* | *torreyana_var._insularis* | C. Greene | 23-Jan-2008 | N33.98398 W120.01992 | N33 59 2.3 W120 01 11.7 | 28 |
| *Chionaspis* | *torreyanae* | D2240D | Paratype | USA | California | Santa Rosa Island | *Pinus* | *torreyana_var._insularis* | C. Greene | 23-Jan-2008 | N33.98398 W120.01992 | N33 59 2.3 W120 01 11.7 | 28 |
